# Magnetic/Zeolitic Imidazolate Framework-67 Nanocomposite for Magnetic Solid-Phase Extraction of Five Flavonoid Components from Chinese Herb *Dicranopteris pedata*

**DOI:** 10.3390/molecules28020702

**Published:** 2023-01-10

**Authors:** Zhiyang Feng, Jiaqing Zhu, Shen Zhuo, Jun Chen, Wenyi Huang, Hao Cheng, Lijun Li, Tai Tang, Jun Feng

**Affiliations:** 1 KingMed College of Laboratory Medicine, Guangzhou Medical University, Guangzhou 510182, China; 2 Department of Medicine, College of Biological and Chemical Engineering, Guangxi University of Science and Technology, Liuzhou 545006, China; 3 State Key Laboratory for Chemistry and Molecular Engineering of Medicinal Resources, Guangxi Normal University, Guilin 541004, China

**Keywords:** magnetic solid-phase extraction, Fe_3_O_4_@ZIF−67, *Dicranopteris pedata*, flavonoids, metal–organic framework materials

## Abstract

A magnetically functionalized Fe_3_O_4_@ZIF−67 metal–organic framework (MOF) was prepared by electrostatic self-assembly using magnetic Fe_3_O_4_ nanoparticles as the core and ZIF−67 as the shell. The composite was characterized by electron microscopy, X-ray diffraction, Fourier- transform infrared spectroscopy, and Brunauer–Emmett–Teller measurements. Magnetic solid-phase extraction (MSPE) was performed on five flavonoids from *Dicranopteris pedata* using Fe_3_O_4_@ZIF−67 as an adsorbent. The developed MSPE method was combined with high-performance liquid chromatography–ultraviolet detection to preconcentrate and separate five flavonoids (rutin, quercitrin, kaempferol-3-O-α-L-rhamnoside, quercetin, and kaempferol) from *Dicranopteris pedata*. The factors affecting the extraction, such as the amount of Fe_3_O_4_@ZIF−67 adsorbent, salt ion concentration in the sample solution, vortex time, type and amount of desorbing solvent, concentration of formic acid to acidify the desorbing solvent, and acetonitrile ratio, were optimized. The developed method showed good linearity over the concentration range of 1.09–70.0 μg∙mL^−1^ for the five flavonoids, with R^2^ values between 0.9901 and 0.9945. The limits of detection and average recoveries for the five flavonoids were in the ranges of 39.5–56.2 ng∙mL^−1^ and 92.2–100.7%, respectively. The method presented herein is simple, efficient, and sensitive; it can be used for enrichment analysis of the five flavonoids in *Dicranopteris pedata*.

## 1. Introduction

*Dicranopteris pedata (Houtt.) Nakai* is a perennial herb found in Dicranopteris Bernh, which belongs to the Gleicheniaceae family. *Dicranopteris pedata* is rich in flavonoids, saponins, and polysaccharides; exhibits strong antioxidant, antibacterial, and anticancer pharmacological properties; and has attracted considerable attention in the biomedical field [1,2,3,4]. To date, flavonoids in *Dicranopteris pedata* have been isolated and analyzed using high-performance liquid chromatography–ultraviolet detection (HPLC-UV) [5] and ultra-performance liquid chromatography–tandem mass spectrometry (UPLC-Q- TOF/MS) [6]. However, owing to the complex composition and low concentration of flavonoids in *Dicranopteris pedata*, pretreatment is required before HPLC-UV or UPLC-Q- TOF/MS analyses to eliminate interferences and improve the sensitivity of the method.

Solid-phase extraction (SPE) is widely used as a pretreatment technique for sample preparation because it uses fewer organic solvents and has higher selectivity than traditional liquid–liquid extraction techniques [7,8]. Metal–organic frameworks (MOFs) are a class of hybrid materials that utilize metal clusters and organic linkers to form crystalline framework structures through coordination bonds and are characterized by high porosity and high specific surface area [9]. Their unique properties make MOFs useful for a wide range of analytical chemistry applications. MOFs have been successfully used as sorbents for SPE and solid-phase microextraction and as stationary phases for gas and liquid chromatography [10,11,12].

Magnetic solid-phase extraction (MSPE) based on magnetic nanoparticles (NPs) has shown great potential for preconcentration and separation owing to the advantages of magnetic separation compared to conventional SPE techniques. First, the separation of the analyte from the solution is accomplished by pure magnetic separation without the need to fill the column, thus avoiding the clogging and inefficient reactivation of conventional SPE columns. For example, Wang et al. [13] synthesized Fe_3_O_4_/MIL-101 (Cr) composites for the determination of non-steroidal anti-inflammatory drugs in complex aqueous solutions, which are more rapid, environment-friendly, and easy to recycle. It also bypasses the bed-filling technique required for column loading [14,15]. Second, the separation occurs rapidly, with a significantly shorter time and lower costs of organic solvents than solid-phase microextraction techniques [16]. Third, the magnetic adsorbent has a larger contact area with the solvent, and thus, the advantages of the adsorption site and large specific surface area can be used more rationally [17]. In addition, the adsorbent can be easily recovered and recycled. Thus, MSPE is now widely used for sample preparation in environmental, food, and biological applications [18,19,20,21].

The subtle coupling of MOFs and MSPE is an interesting and important technique for SPE applications considering the unique properties of MOFs, such as high porosity and high specific surface area. However, owing to the limited magnetization strength of MOFs, magnetic functionalization of the material is necessary. Therefore, it is expected that magnetized MOFs can be applied as adsorbents for SPE to achieve better sample pretreatment results when analyzing plant samples with complex compositions, many interfering substances, and low concentrations of the substances to be measured [22].

In this work, we prepared magnetically functionalized Fe_3_O_4_@ZIF−67 by electrostatic self-assembly using Fe_3_O_4_ magnetic NPs as the core. The material was used as a solid-phase extraction sorbent for the effective preconcentration of flavonoids in the Chinese medicine *Dicranopteris pedata*. A detailed evaluation of the potential factors affecting the adsorption performance of Fe_3_O_4_@ZIF−67 was performed to determine the full potential of MSPE of flavonoids in *Dicranopteris pedata*. The adsorption mechanism of Fe_3_O_4_@ZIF−67 for flavonoids was explored. Finally, a new MSPE-HPLC-UV coupling method was developed for the separation and determination of rutin, quercitrin, kaempferol-3-O-α-L-rhamnoside, quercetin, and kaempferol. This method was successfully applied for the simultaneous separation and determination of the five flavonoids in *Dicranopteris pedata*.

## 2. Results and Discussion

### 2.1. Selection of Adsorbents for Magnetic MOFs

Seven different materials were used for the adsorption of flavonoid components from *Dicranopteris pedata* and analyzed by HPLC-UV. As shown in Figure S1, Fe_3_O_4_@ZIF−4, Fe_3_O_4_@MOF−74, and Fe_3_O_4_@ZIF−67 showed high adsorption and removal capacities, whereas Fe_3_O_4_@ZIF−67 showed the highest adsorption and the best removal effect. ZIF−4 has some adsorption capacity for flavonoids owing to its ability to form hydrogen bonds because of the large number of imidazole ligand-bridging structures. Therefore, ZIF−4 is more likely to adsorb flavonoids with more hydroxyl groups. It is believed that the larger molecular structure of flavonoids affects their diffusion into the MOF pores, which is one of the reasons for the limited adsorption capacity of ZIF−4 for flavonoids [23]. In addition, the small size of the pore cage cavity of ZIF−4, approximately 3.3 Å [24], also limits the diffusion of compounds into the material. The cavity cage size of ZIF−67 (10 Å) is significantly larger than that of ZIF−4, which explains the lower adsorption efficiency of ZIF−4 compared to ZIF−67. Unlike ZIF−67, the positive surface charge of the MIL−101 series materials decreased with increasing the pH [25], and the adsorption capacity decreased continuously, whereas the pH of the *Dicranopteris pedata* sample was neutral and unsuitable for the adsorption environment of the MIL-101 series. The luminal pore cage size of MOF−74 (Co-Zn) is 11 Å, and although it does not limit the diffusion of flavonoids within the pore channel, its surface charge is lower than that of the other materials of the ZIF series [26], which explains the lower adsorption of MOF−74 (Co-Zn). Therefore, Fe_3_O_4_@ZIF−67 was used as a solid-phase adsorbent for flavonoids in *Dicranopteris pedata*.

### 2.2. Characterization of Fe_3_O_4_@ZIF−67

**Figure 1:**
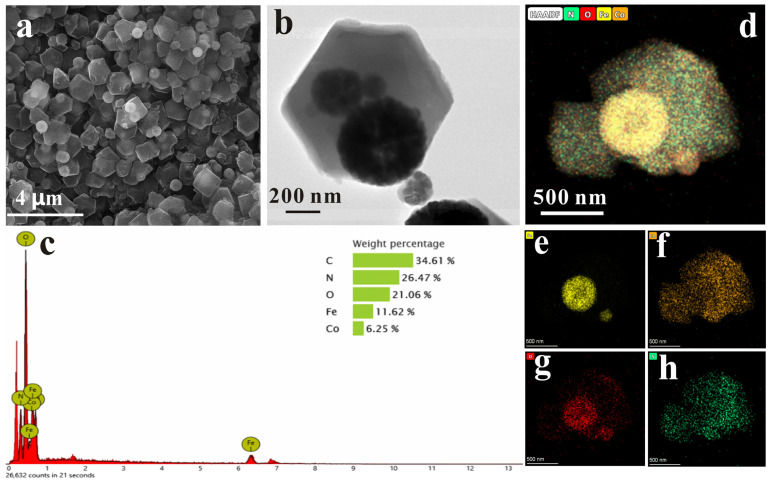
The SEM (**a**) and TEM images (**b**) of Fe_3_O_4_@ZIF−67. (**c**) The energy-dispersive X-ray spectroscopy (EDS) diagram of Fe_3_O_4_@ZIF−67. (**d**) The distribution of each element in the Fe_3_O_4_@ZIF−67. (**e**–**h**) EDS elemental mapping of Fe, Co, O, and N of the Fe_3_O_4_@ZIF−67.

The morphology of Fe_3_O_4_@ZIF−67 was characterized using scanning electron microscopy (SEM) and transmission electron microscopy (TEM) (Figure 1a,b). The SEM images show the morphology of ZIF−67 as a dodecahedron with well-defined edges. Figure 1B shows the TEM characterization of Fe_3_O_4_@ZIF−67, from which it is observed that the surface of ZIF−67 was modified by aggregated Fe_3_O_4_ NPs with clearly visible edges, indicating that the Fe_3_O_4_ NPs successfully magnetized ZIF−67. Figure 1c illustrates that the Fe_3_O_4_@ZIF−67 composite consists of the elements C, N, O, Fe, and Co and gives the percentage of each element, which are the basic elements that make up Fe_3_O_4_@ZIF−67. Figure 1e,f,g,h show the distribution of Fe, Co, N, and C, respectively, indicating a uniform distribution of each element during the synthesis.

**Figure 2:**
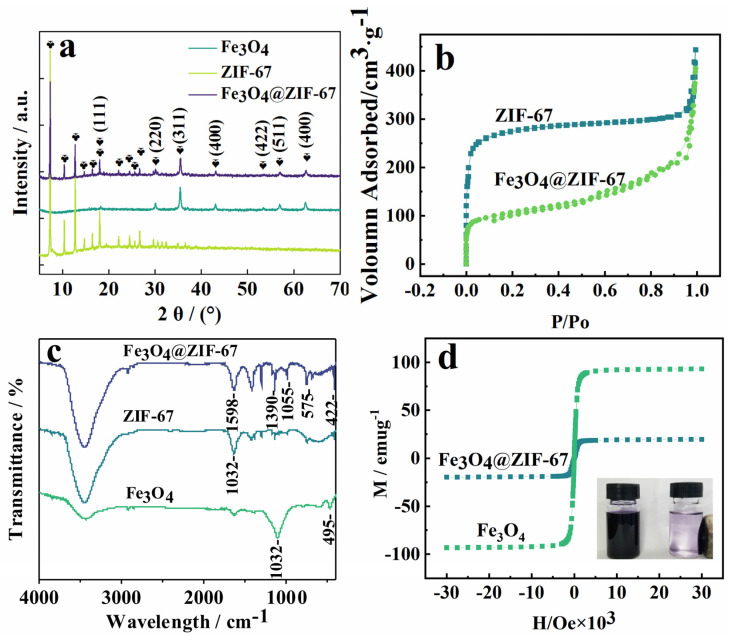
(**a**) XRD patterns of Fe_3_O_4_@ZIF−67, ZIF−67, and Fe_3_O_4_. (**b**) Nitrogen adsorption– desorption isotherms of ZIF−67 and Fe_3_O_4_@ZIF−67 at 77K. (**c**) FT-IR spectra of Fe_3_O_4_@ZIF−67, ZIF−67, and Fe_3_O_4_. (**d**) Hysteresis loops of Fe_3_O_4_ and Fe_3_O_4_@ZIF−67; inset, photographs of Fe_3_O_4_@ZIF−67 before and after magnetic separation with an external magnet.

Figure 2a shows the X-ray diffraction (XRD) patterns of Fe_3_O_4_, ZIF−67, and Fe_3_O_4_@ZIF−67. The presence of characteristic peaks of ZIF−67 and Fe_3_O_4_ in the XRD patterns of Fe_3_O_4_@ZIF−67 confirms the formation of Fe_3_O_4_@ZIF−67. The characteristic peaks of Fe_3_O_4_@ZIF−67 at 18.20° (111), 30.22° (220), 35.68° (311), 43.28° (400), 53.68° (422), 57.18° (511), and 62.76° (440) correspond to the characteristic peaks of Fe_3_O_4_ NPs, indicating that the crystal structure of Fe_3_O_4_@ZIF−67 is intact and there was no serious crystallographic disruption in ZIF−67 by the incorporation of Fe_3_O_4_ during the synthesis.

The porosities of ZIF−67 and Fe_3_O_4_@ZIF−67 were measured using N_2_ adsorption isotherms at 77 K (Figure 2b). The specific surface area of ZIF−67 calculated using the Brunauer–Emmett–Teller (BET) method was 890.307 m^2^∙g^−1^, with a mesopore pore size of 6.3030 nm. The specific surface area of Fe_3_O_4_@ZIF−67 was 379.517 m^2^∙g^−1^ with a mesopore size of 8.7226 nm. Compared to the BET surface area of pure ZIF−67, that of Fe_3_O_4_@ZIF−67 was slightly smaller owing to the presence of the non-porous Fe_3_O_4_ core but was still a large specific surface area.

The Fourier-transform infrared (FT-IR) spectra of Fe_3_O_4_, ZIF−67, and Fe_3_O_4_@ZIF−67 were characterized using FT-IR spectroscopy (Figure 2c). The characteristic band at 422 cm^−1^ in the FT-IR spectrum of Fe_3_O_4_@ZIF−67 was attributed to Co-N stretching vibrations. The peaks at 1055 and 1392 cm^−1^ confirmed the presence of benzene rings. The peaks at 575 cm^−1^ were attributed to Fe-O-Fe bonds in the Fe_3_O_4_ particles. The characteristic band at 1350–1500 cm^−1^ can be attributed to the stretching vibration of the imidazole ring. The characteristic band at 1590 cm^−1^ is consistent with the C=N stretching vibrations in ZIF−67. The FT-IR spectra of Fe_3_O_4_@ZIF−67 showed characteristic peaks of ZIF−67, indicating that Fe_3_O_4_ NPs were successfully loaded onto ZIF−67, which is consistent with the XRD and SEM results.

As shown in Figure 2d, the saturation magnetization values of Fe_3_O_4_ and Fe_3_O_4_@ZIF−67 were classified as 97.16 and 19.79 emu∙g^−1^, respectively. The magnetic properties of Fe_3_O_4_@ZIF−67 decreased with the encapsulation of ZIF−67 but were still good (inset of Figure 2d). The relatively high saturation magnetization values of Fe_3_O_4_@ZIF−67 make it susceptible to magnetic fields and easy to separate from aqueous solutions. The absence of significant hysteresis, remanence, and coercivity of the hysteresis line illustrates the superparamagnetic nature of Fe_3_O_4_@ZIF−67, which is crucial for magnetic extraction.

The zeta potential of the Fe_3_O_4_@ZIF−67 surface was measured. As shown in Figure S2, the positive charge of Fe_3_O_4_@ZIF−67 gradually increased with increasing pH, reaching a maximum value of 35 mV at pH 8. When crossing the isotropic potential (PI = 9), Fe_3_O_4_@ZIF−67 changed from a positive to a negative surface charge.

### 2.3. Optimization of MSPE Extraction Conditions

#### 2.3.1. Selection of Desorbing Solvent

**Figure 3:**
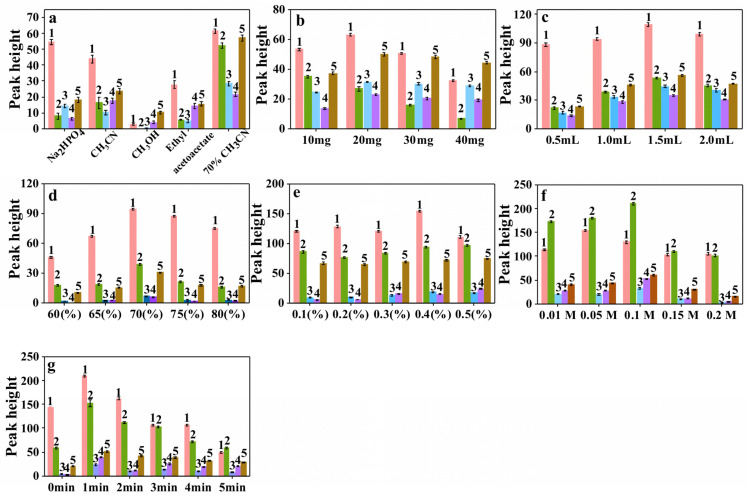
The effect of (**a**) desorbing solvent, (**b**) amount of Fe_3_O_4_@ZIF−67, (**c**) desorbing solvent volume, (**d**) the proportion of acetonitrile in the desorbing solvent, (**e**) the concentration of formic acid to acidify the desorbing solvent, (**f**) the salt ion concentration in the sample solution, and (**g**) vortex time on MSPE performance (error bars indicate standard deviations from three measurements). Sample: five flavonoids’ mixed standard solutions; 1, kaempferol-3-O-α-L- rhamnoside; 2, quercitrin; 3, rutin; 4, quercetin; 5, kaempferol.

The surface of Fe_3_O_4_@ZIF−67 was positively charged at a pH below 8 (Figure S2). Flavonoids rich in hydroxyl groups are adsorbed onto Fe_3_O_4_@ZIF−67 owing to electrostatic forces upon entering the pore cage of Fe_3_O_4_@ZIF−67; therefore, a suitable desorbing solvent is critical. A mixture of methanol, acetonitrile, ethyl acetate, phosphate buffer, and 70% acetonitrile solution was evaluated for the desorption of flavonoids from Fe_3_O_4_@ZIF−67. Figure 3a compares the performance of the five solvents. Under the same extraction and elution conditions, the five flavonoids showed the highest peak heights in the chromatogram when a 70% solution of acetonitrile was used as the desorbing solvent. This demonstrated the superior desorption performance of the 70% acetonitrile solution, which was therefore chosen as the desorbing solvent for the remaining experiments.

#### 2.3.2. Effect of the Amount of Fe_3_O_4_@ZIF−67

The effect of the amount of Fe_3_O_4_@ZIF−67 (10, 20, 30, and 40 mg) on the extraction efficiency was investigated. As shown in Figure 3b, initially, the peak heights of the five flavonoids increased with the amount of Fe_3_O_4_@ZIF−67. The peak heights of most of the flavonoids reached a maximum (i.e., optimum extraction efficiency) when the amount of Fe_3_O_4_@ZIF−67 was 20 mg and started to decrease at 30 mg. In general, an increase in the amount of sorbent will improve the extraction capacity. However, in the elution phase, the more sorbent that is involved, the more difficult it is for the analytes to be eluted from the sorbent. Even the latter may be more prominent, which leads to the result that increasing the amount of sorbent decreases the recovery [27]. Therefore, 20 mg of Fe_3_O_4_@ZIF−67 was chosen for subsequent experiments.

#### 2.3.3. Optimization of the Desorbing Solvent Volume 

The effect of the desorbing solvent volume, set to 0.5, 1.0, 1.5, and 2 mL, on the desorption performance was investigated. As shown in Figure 3c, the amounts of eluted flavonoids increased as the volume of the desorbing solvent increased. At 1.5 mL, the peak height of the flavonoids reached the maximum, but the interfering substances also increased (Figure S3). Considering the desorption performance and interfering impurities, 1.0 mL was chosen as the optimum volume for the desorbing solvent.

#### 2.3.4. Effect of the Proportion of Acetonitrile in the Desorbing Solvent 

Different ratios of desorbing solvents were examined. As shown in Figure 3d, the desorption performance was analyzed at acetonitrile ratios of 60, 65, 70, 75, and 80%. The experimental results showed that the best desorption performance was achieved with 70% acetonitrile, which was thus chosen as the optimum eluent ratio.

#### 2.3.5. Effect of Formic Acid Concentration to Acidify Desorbing Solvent

The concentration of formic acid used to acidify the desorbing solvent had a strong influence on the desorption performance. Maintaining the desorbing solvent under acidic conditions can protect flavonoids from oxidative degradation [28] and improve the extraction efficiency of the substances to be measured. Additionally, the acidity influences how much the analyte dissociates in the solvent, which changes the analyte’s surface charge and, consequently, the electrostatic adsorption between the analyte and the sorbent. Therefore, we investigated the effect of acidifying the desorbing solvent on the extraction efficiency by using different concentrations of formic acid (concentration range of 0.1–0.5%). As shown in Figure 3e, with the increase in formic acid concentration, flavonoids were continuously desorbed from the sorbent and the corresponding peak heights increased. However, the chromatogram of the *Dicranopteris pedata* samples showed that the peak heights of the interfering substance peaks were also increasing (Figure S4). Considering the extraction efficiency of the substances to be evaluated and the influence of interfering substances, 0.3% was selected as the optimal concentration of formic acid to acidify the desorbing solvent for the remaining experiments.

#### 2.3.6. Effect of Salt Ion Concentration in the Sample Solution

The effect of the ionic strength of the sample on the extraction efficiency of Fe_3_O_4_@ZIF−67 was investigated by varying the concentration of NaAc in the mixed standard solutions of the five flavonoids (Figure 3f) at 0.01, 0.05, 0.1, 0.15, and 0.2 M. The highest extraction efficiency for each substance to be evaluated was achieved at 0.1 M. Above that, the efficiency decreased. Therefore, the optimum salt ion concentration was determined to be 0.1 M. This indicates that the adsorption of Fe_3_O_4_@ZIF−67 can be improved by tuning the ionic strength; however, a high salt ion concentration affects the electrostatic interaction, making the adsorption rate less efficient than that at low ionic concentrations [29].

#### 2.3.7. Effect of Vortex Time on Adsorption Capacity 

For MSPE, vortex is a time-dependent process. Therefore, the effect of vortex extraction times of 0, 1, 2, 3, 4, and 5 min on the extraction efficiency of Fe_3_O_4_@ZIF−67 was investigated. As shown in Figure 3g, the extraction efficiency was optimal at a vortex extraction time of 1 min, which was thus elected as the optimal vortex extraction time. This may be because the dispersion extraction mode increases the interfacial area between the sorbent and the target sample solution, thus facilitating mass transfer and allowing the adsorption to reach equilibrium rapidly [27].

In summary, the optimal conditions for MSPE are as follows: 70% acetonitrile solution as the desorbing solvent, Fe_3_O_4_@ZIF−67 at an amount of 20 mg, desorbing solvent at a volume of 1.0 mL, proportion of acetonitrile in the desorbing solvent of 70%, concentration of formic acid to acidify the desorbing solvent of 0.3%, concentration of salt ions in the sample solution of 0.1 M, and vortex time of 1.0 min.

### 2.4. Stability and Reusability of Fe_3_O_4_ @ZIF−67

The stability of Fe_3_O_4_@ZIF−67 was examined at 7, 14, 21, and 28 days under optimal MSPE conditions. Changes in the extractive properties of the materials were recorded. Three mixed standard solutions of the five flavonoid compounds were prepared in parallel, and the same Fe_3_O_4_@ZIF−67 was used for MSPE-HPLC-UV measurements on days 0, 7, 14, 21, and 28, respectively, with three parallel measurements for each sample. The recoveries of Fe_3_O_4_@ZIF−67 for each flavonoid at different times were calculated using Equation (1).

(1) $$R=\frac{A_{n}}{A_{0}}\times 100\%$$

where *R* is the recovery of each flavonoid from Fe_3_O_4_@ZIF−67 on days 7, 14, 21, and 28; *A*_0_ is the peak area of each flavonoid on day 0; and *A_n_* is the peak area of each corresponding flavonoid on day *n* (*n* = 0, 7, 14, 21, 28).

**Figure 4:**
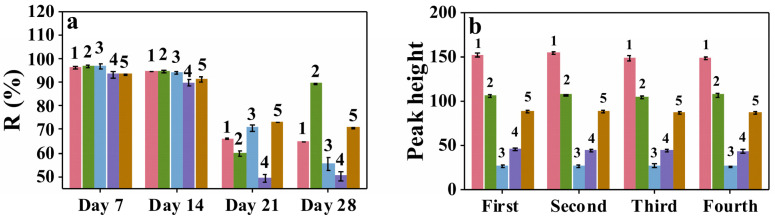
(**a**) Stability of Fe_3_O_4_@ZIF−67 for different storage times. (**b**) Recycling of Fe_3_O_4_@ZIF−67 for flavonoids extraction (error bars indicate standard deviations from three measurements). Sample: five flavonoids’ mixed standard solutions; 1, kaempferol-3-O-α-L-rhamnoside; 2, quercitrin; 3, rutin; 4, quercetin; 5, kaempferol.

As shown in Figure 4a, the *R* value of all five flavonoids was over 90% on days 7 and 14. However, after days 21 and 28, *R* rapidly decreased to below 80%, indicating that the stability of Fe_3_O_4_@ZIF−67 decreased rapidly with increasing storage time. Therefore, Fe_3_O_4_@ZIF−67 is stable enough to use until 14 days of storage.

The reusability of Fe_3_O_4_@ZIF−67 was evaluated under optimized conditions. The sorbent was washed and dried with methanol before proceeding to the next cycle. As shown in Figure 4b, the extraction efficiency of the sorbent remained almost unchanged (RSD ≤ 4.2%) after four consecutive adsorption–desorption cycles. This phenomenon indicates that the sorbent has good reusability.

### 2.5. Validation of Method

The established MSPE-HPLC-UV method was examined for five flavonoids’ mixed standard solutions in terms of precision, linearity, the limit of detection (LOD), the limit of quantification (LOQ), and accuracy. The performance of this method was evaluated under optimal conditions following the International Conference on Harmonisation document on validation methodology [30] and the procedure described by Rambla-Alegre et al. [31]. As shown in Table 1, the determination coefficients (R^2^) ranged from 0.9901 to 0.9945 with good linearity. The LOD (S/N = 3) and LOQ (S/N = 10) were calculated according to the 3σ/m criterion proposed by the IUPAC, with σ being the standard deviation of the blanks and m being the slope of the calibration plot. The obtained values were in the range of 39.5–56.2 ng∙mL^−1^ and 130.5–147.2 ng∙mL^−1^ for LOD and LOQ, respectively, indicating the high sensitivity of the method.

### 2.6. Method Application

**Figure 5:**
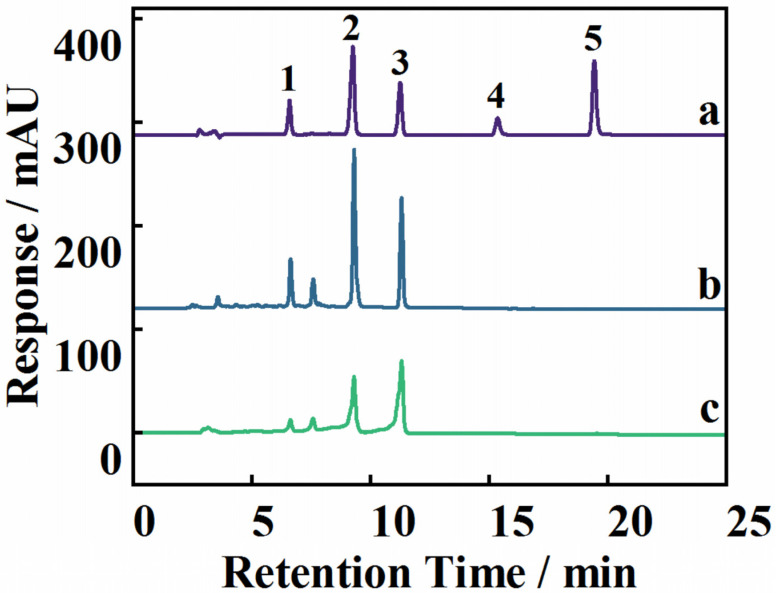
The chromatogram of five flavonoids’ mixed standards (a), the *Dicranopteris pedata* samples after MSPE (b), and the *Dicranopteris pedata* samples without MSPE (c). Note: 1, rutin; 2, quercitrin; 3, kaempferol-3-O-α-L-rhamnoside; 4, quercetin; 5, kaempferol.

The MSPE–HPLC-UV method was applied to the extraction and analysis of flavonoids from *Dicranopteris pedata* under optimal extraction conditions to evaluate the practicality of the developed method in real samples. Figure 5 shows the chromatograms of the *Dicranopteris pedata* samples before and after MSPE. The interfering substances were greatly reduced in the samples after MSPE, thereby reducing the influence on the target analytes. The quantitative results of the five flavonoids are shown in Table 2. Kaempferol-3-O-α-L-rhamnoside, quercitrin, and rutin were enriched by 1.18-, 3.11-, and 6.34-fold, respectively. Quercetin and kaempferol were not found in the *Dicranopteris pedata* samples. The precision and accuracy of the proposed method were verified by analyzing spiked samples at low, medium, and high concentration levels. Relative recoveries and relative standard deviations (RSDs) were used to express the precision and accuracy of the proposed method. The relative recoveries of the five flavonoids were in the range of 91.2–99.9%, with RSDs in the range of 0.24–2.2% (Table 2), indicating that the method is reliable and reproducible and highlighting its feasibility and applicability.

By comparison with other reported methods for the determination of flavonoids (Table 3), it can be seen that the developed MSPE-HPLC-UV method based on the Fe_3_O_4_@ZIF−67 adsorbent also has good sensitivity and high recovery. Moreover, the method is simple and convenient due to the fast magnetic separation during sample enrichment.

## 3. Materials and Methods

### 3.1. Materials

*Dicranopteris pedata* was harvested from the Sanmenjiang National Forest Park, Liuzhou, Guangxi. All reagents and chemicals used were of analytical grade. Methanol and acetonitrile (HPLC-grade) were obtained from Merck (Darmstadt, Germany). Zinc nitrate hexahydrate (Zn(NO_3_)_2_·6H_2_O), cobalt nitrate hexahydrate (Co(NO_3_)_2_·6H_2_O), chromium oxide nonahydrate (Cr(NO_3_)_3_·9H_2_O), imidazole, 2-methylimidazole (Mim), terephthalic acid, 2-amino terephthalic acid, and 2,5-dihydroxy terephthalic acid were purchased from Shanghai Aladdin Biochemical Technology Co. Ltd. Rutin (95%), kaempferol (97%), kaempferol-3-O-α-L-rhamnoside (98%), quercitrin (98%), and quercetin (95%) were purchased from Shanghai Maclean Biochemical Technology Co. Ltd. Sodium phosphate monobasic dihydrate (NaH_2_PO_4_·2H_2_O), glacial acetic acid, sodium hydroxide, iron (III) chloride hexahydrate (FeCl_3_·6H_2_O), sodium acetate (NaAc), ethylene glycol, absolute ethanol, ammonia (25%), N,N-dimethylformamide (DMF), chloroform (CHCl_3_), ethyl orthosilicate (TEOS), and ethyl acetate were obtained from Guangdong Guanghua (Guangdong, China). The deionized (DI) water (18.2 MΩ·cm) used in our experiments was produced using a UPH-1 V-20 T Houpu series ultrapure water machine (Sichuan Houpu Super Pure Technology Co., Ltd., Chengdu, China). 

### 3.2. Instrumentation and Chromatographic Conditions 

The morphology and size distribution of the particles were observed using scanning electron microscopy (SEM; Hitachi S-4800, Tokyo, Japan) and transmission electron microscopy (TEM; JEOL JEM 2100, Tokyo, Japan). The elemental analysis chart was measured using energy-dispersive X-ray spectroscopy (EDS; Oxford, UK). Powder X-ray diffraction (XRD; Bruker D8A A25, Bruker, Germany) and Fourier-transform infrared (FT-IR) spectroscopy (Frontier, FL, USA) were performed to characterize the material structure. The specific surface area and pore size distribution of the materials were measured using Brunauer–Emmett–Teller (BET) measurements with an ASAP-2460 gas adsorption instrument (Micromeritics, USA). The zeta potential of ZIF−67 was measured using a Zetasizer Nano ZS (NanoZS90; Malvern, UK). All hysteresis loops were collected using an MPMS-XL SQUID magnetometer and a vibrating sample magnetometer (VSM) (Quantum Design, Caledonia, IL, USA). 

The chromatographic system consisted of an Agilent 1260 HPLC with a variable wavelength detector (Agilent Technologies, Santa Clara, CA, USA). All analyses were performed on a Pntulips TMBP C18 column (4.6 mm × 250 mm, 5 μm). The mobile phase consisted of phase A (acetonitrile with 1% formic acid) and phase B (an aqueous solution with 0.2% formic acid). The flow rate, UV detection wavelength, injection volume, and column temperature were 1 mL∙min^−1^, 365 nm, 20 µL, and 30 °C, respectively. All analytes were separated using gradient elution, whose conditions are shown in Table S1.

### 3.3. Preparation of Fe_3_O_4_ NPs

Fe_3_O_4_ NPs were prepared according to a previous report with slight modifications [36]. Certain amounts of FeCl_3_·6H_2_O and 8.46 g of sodium acetate were dissolved in 64 mL of ethylene glycol. After stirring for 30 min, the mixed solution was transferred to an autoclave with a PTFE liner and heated at 220 °C for 24 h. After cooling and separation under an applied magnetic field, the prepared products were alternately washed three times with deionized water and ethanol. Finally, the product was dried in a vacuum drying oven at 60 °C for 12 h to obtain Fe_3_O_4_ NPs.

### 3.4. Preparation of Fe_3_O_4_@ZIF−67 

Fe_3_O_4_@ZIF−67 was prepared according to the literature protocol [37] with slight modifications. In brief, 0.02 g of prepared Fe_3_O_4_ NPs and 0.5730 g of Co(NO_3_)_2_·6H_2_O were dissolved in methanol (7.5 mL) to obtain solution A. Additionally, 0.6512 g of 2-methylimidazole was dissolved in methanol (7.5 mL) and sonicated for 5 min to obtain solution B. Solution A was then mixed with solution B for 10 min and left to age for 12 h. The product was separated by an applied magnetic field, dried in a vacuum drying oven at 60 °C for 20 min, and ground for subsequent experiments.

The details of the preparation procedure for Fe_3_O_4_@MIL-101 (Fe), Fe_3_O_4_@MIL-101 (Cr), Fe_3_O_4_@ZIF-4, Fe_3_O_4_@IRMOF-3, Fe_3_O_4_@Zn-MOF-74, and Fe_3_O_4_@Co-MOF-74 can be found in the Supplementary Materials S1 section.

### 3.5. Sample Preparation

The freshly harvested *Dicranopteris pedata* was dried in a drying oven at 105 °C. The stems and leaves were crushed and passed through a 20-mesh sieve. Then, 1.0 g of the sieved powder was dissolved in 40 mL of 80% ethanol at 85 °C for 2 h with reflux condensation. The resulting extract was concentrated to 10 mL, diluted 10 times with absolute ethanol, and used as a sample solution for subsequent experiments.

### 3.6. MSPE Procedure

**Figure 6:**
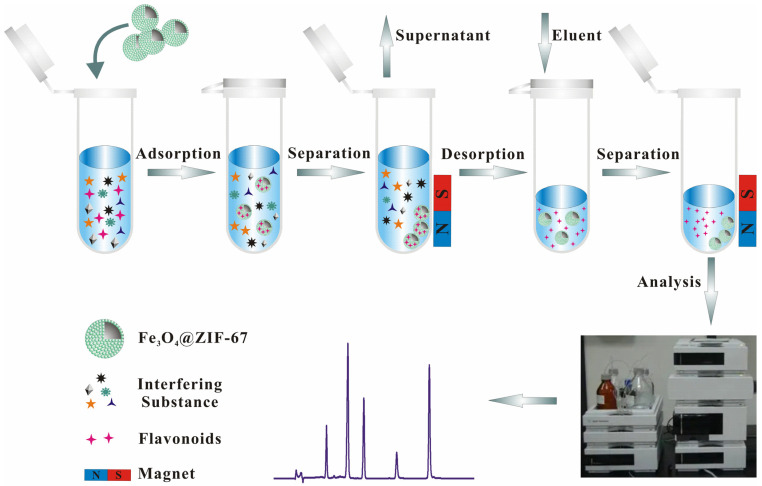
Schematic illustration of the procedure of Fe_3_O_4_@ZIF−67-based MSPE.

The extraction procedure for the five flavonoids using the magnetic MOF is shown in Figure 6. First, a certain amount of the prepared magnetic MOF was dispersed in the *Dicranopteris pedata* sample solution. The suspension was vortexed and thoroughly shaken. The individual magnetic MOFs were then separated from the solution under an applied magnetic field and the supernatant was discarded. Next, the adsorbed analytes were eluted from the magnetic MOFs with 1 mL of eluent by sonication for 2 min and repeated twice. The eluate was then separated by an applied magnetic field, dried with N_2_, and dissolved in 200 μL of acetonitrile. The resulting product was filtered through a 0.22-micrometer filter before HPLC-UV analysis.

## 4. Conclusions

In this study, magnetically functionalized Fe_3_O_4_@ZIF−67 was prepared by electrostatic self-assembly and overgrowth to be used as an SPE sorbent for the extraction of flavonoids from the traditional Chinese medicine *Dicranopteris pedata*. A method was developed for the determination of the five flavonoids in *Dicranopteris pedata* using MSPE-HPLC-UV. The extraction conditions, including the amount of Fe_3_O_4_@ ZIF−67, the type and amount of the desorbing solvent, the concentration of formic acid to acidify the desorbing solvent, the ratio of acetonitrile of the desorbing solvent, the vortex extraction time, and the ionic strength of the sample, were optimized, resulting in considerable flavonoid recoveries. The method exhibited low detection limits, high precision, high accuracy, simple operation, and advantageous results. It can be used for the analysis of flavonoids in other Chinese herbal medicines.

## Figures and Tables

**Table 1:** Calibration curves, LODs, and LOQs of five flavonoids.

Compound	Linearity Equation	Determination Coefficient (R^2^)	Linearity Range (μg∙mL^−1^)	Intra-Day RSD (%, *n* = 3)	Inter-Day RSD (%, *n* = 3)	LOD (ng∙mL^−1^)	LOQ (ng∙mL^−1^)
Rutin	y = 7.7124x − 18.831	0.9922	4.38–70.00	3.6	4.9	41.4	132.4
Quercitrin	y = 10.994x + 118.94	0.9913	2.34–37.50	2.2	3.8	56.2	147.2
Kaempferol-3-O-α-L-rhamnoside	y = 4.8385x + 18.599	0.9901	1.25–20	3.0	5.8	40.2	131.2
Quercetin	y = 12.554x + 5.8314	0.9945	1.09–17.50	2.8	4.1	39.5	130.5
Kaempferol	y = 18.289x + 68.642	0.9921	2.03–32.50	4.0	4.1	42.8	133.8

**Table 2:** Recovery results of five flavonoids (*n* = 3).

Compound	Sample Content (μg∙mL^−1^) ± SD	Added (μg∙mL^−1^)	Detected (μg∙mL^−1^) ± SD	Recovery (%)	RSD (%)	Average Recovery (%)
Rutin	18.9 ± 0.33	17.5	32.3 ± 0.84	88.8		
		8.7	26.1 ± 0.80	94.5	2.2	92.2
		4.3	21.7 ± 0.50	93.3		
Quercitrin	19.6 ± 0.26	12.5	33.5 ± 0.71	104.7		
		6.2	25.8 ± 0.68	99.8	1.4	100.7
		3.1	22.2 ± 1.31	96.2		
Kaempferol-3-O-α-L-rhamnoside	3.1 ± 0.07	5.0	8.1 ± 0.18	99.6		
		2.5	5.4 ± 0.15	96.8	1.9	95.6
		1.2	3.9 ± 0.10	90.0		
Quercetin	0	5.0	4.8 ± 0.13	95.7		
		2.5	2.3 ± 0.13	92.5	1.8	92.6
		1.3	1.1 ± 0.08	89.7		
Kaempferol	0	10.4	10.0 ± 0.16	96.5		
		5.2	5.1 ± 0.24	98.8	0.2	96.3
		2.6	2.4 ± 0.22	93.5		

**Table 3:** Comparison of various methods for the determination of flavonoids.

Flavonoids	Adsorbent	Analytical Technique	Matrix	LOD (ng∙mL^−1^)	Recovery (%)	Reference
Rutin, quercetin, and kaempferol	OasisTM HLB	SPE-UHPLC-PDA	Wine extract	10.0–59.0	63.0–114.0	[32]
Rutin, quercetin, and kaempferol	TiO_2_ NPs and diatomaceous earth	Multi-step matrix solid-phase dispersion (MSPD)- capillary LC and LC-MS/MS	Residual brewing yeast	7.0–11.0	—	[33]
Rutin, quercetin, and kaempferol	—	Ultrasound-assisted extraction (UAE)-HPLC-UV	Flos Sophorae Immaturus	2000.0–4000.0	97.7–99.3	[34]
Rutin, quercetin, and kaempferol	Supelco LC-18	SPE-LC-MS/MS	Red onion, orange peel, and honey	39.0–76.0	86.0–114.0	[35]
Rutin, quercitrin, kaempferol-3-O-α-L-rhamnoside, quercetin, and kaempferol	Fe_3_O_4_@ZIF−67	MSPE-HPLC-UV	*Dicranopteris pedata*	39.5–56.2	92.2–100.7	This work

## Data Availability

Available data are presented in the manuscript.

## Supplementary Materials

The following supporting information can be downloaded at: https://www.mdpi.com/article/10.3390/molecules28020702/s1, Figure S1: The extraction performance of seven different magnetic MOFs; Figure S2: The zeta potential (b) for different pH values; Figure S3: Chromatograms of Dicranopteris pedata samples after desorption with different desorbing solvent volumes; Figure S4: Chromatograms of Dicranopteris pedata samples after desorption with different formic acid concentrations to acidify the desorbing solvent—1, rutin; 2, quercitrin; 3, kaempferol-3-O-α-L-rhamnoside; Table S1: The gradient elution conditions of HPLC-UV. [23,26,38,39,40].

## References

[1] Lai H.Y., Lim Y.Y., Tan S.P. (2009). Antioxidative, Tyrosinase Inhibiting and Antibacterial Activities of Leaf Extracts From Medicinal Ferns. Biosci. Biotechnol. Biochem..

[2] Goswami H.K., Sen K., Mukhopadhyay R. (2016). Pteridophytes: Evolutionary boon as medicinal plants. Plant Genet. Resour. C.

[3] Eugene C., Kim H.W., Baek H., Kim H., Jo G.U., Park S., Oh C., Deuk-sil O., Kim J. (2020). A study on the antioxidant and anticancer activities of *Dicranopteris pedeta* aqueous extract. J. Adv. Eng. Technol..

[4] Baharuddin A.A., Roosli R.A., Zakaria Z.A., Tohid S.F.M. (2021). *Dicranopteris* linearis A potential medicinal plant with anticancer properties. Boletín Latinoam. Y Del Caribe De Plantas Med. Y Aromáticas.

[5] Zakaria Z.A., Kamisan F.H., Kek T.L., Salleh M.Z. (2020). Hepatoprotective and antioxidant activities of *Dicranopteris* linearis leaf extract against paracetamol-induced liver intoxication in rats. Pharm. Biol..

[6] Yang W., Ye M., Qiao X., Wang Q., Bo T., Guo D. (2012). Collision-induced Dissociation of 40 Flavonoid Aglycones and Differentiation of the Common Flavonoid Subtypes Using Electrospray Ionization Ion-trap Tandem Mass Spectrometry and Quadrupole Time-of-Flight Mass Spectrometry. Eur. J. Mass Spectrom..

[7] Li P., Li M., Yue D., Chen H. (2022). Solid-phase extraction methods for nucleic acid separation. A review. J. Sep. Sci..

[8] Kotova A.A., Thiebaut D., Vial J., Tissot A., Serre C. (2022). Metal-organic frameworks as stationary phases for chromatography and solid phase extraction: A review. Coord. Chem. Rev..

[9] Muguruza A.R., De Luis R.F., Iglesias N., Bazan B., Urtiaga M.K., Larrea E.S., Fidalgo-Marijuan A., Barandika A.G. (2020). Encapsulation of beta-alanine model amino-acid inzirconium (IV) metal (IV) metal organic frameworks: Defect engineering to improve host guest interactions. J. Inorg. Biochem..

[10] Jiang Y., Ma P., Piao H., Qin Z., Tao S., Sun Y., Wang X., Song D. (2019). Solid-phase microextraction of triazine herbicides via cellulose paper coated with a metal-organic framework of type MIL-101(Cr), and their quantitation by HPLC-MS. Microchim. Acta.

[11] Manousi N., Zachariadis G.A., Deliyanni E.A. (2021). On the use of metal-organic frameworks for the extraction of organic compounds from environmental samples. Environ. Sci. Pollut. Res..

[12] Si T., Lu X., Zhang H., Wang S., Liang X., Guo Y. (2022). Metal-organic framework-based core-shell composites for chromatographic stationary phases. TrAC Trends Anal. Chem..

[13] Wang T., Liu S., Gao G., Zhao P., Lu N., Lun X., Hou X. (2017). Magnetic Solid Phase Extraction of Non-steroidal Anti-inflammatory Drugs From Water Samples Using a Metal Organic Framework of Type Fe_3_O_4_/MIL-101(Cr), and Their Quantitation By UPLC-MS/MS. Microchim. Acta.

[14] Plotka-wasylka J., Szczepańska N., De la Guardia M., Namiesnik J. (2015). Miniaturized Solid-phase Extraction Techniques. TrAC Trends Anal. Chem..

[15] Li N., Jiang H., Wang X., Wang X., Xu G., Zhang B., Wang L., Zhao R., Lin J. (2018). Recent Advances in Graphene-based Magnetic Composites for Magnetic Solid-phase Extraction. TrAC Trends Anal. Chem..

[16] Suleiman J., Hu B., Peng H., Huang C. (2009). Separation/preconcentration of Trace Amounts of Cr, Cu and Pb in Environmental Samples By Magnetic Solid-phase Extraction with Bismuthiol-ii-immobilized Magnetic Nanoparticles and Their Determination By ICP-OES. Talanta.

[17] Li X., Zhu G., Luo Y., Yuan B., Feng Y. (2013). Synthesis and Applications of Functionalized Magnetic Materials in Sample Preparation. TrAC Trends Anal. Chem..

[18] Boontongto T., Burakham R. (2020). Simple magnetization of Fe_3_O_4_/MIL-53(Al)-NH_2_ for a rapid vortex-assisted dispersive magnetic solid-phase extraction of phenol residues in water samples. J. Sep. Sci..

[19] Xu Y., Li Z., Yang H., Ji X., Zhang H., Li Y., Zhou M., Wang J., Qian M. (2022). A magnetic solid phase extraction based on UiO-67@GO@Fe_3_O_4_ coupled with UPLC-MS/MS for the determination of nitroimidazoles and benzimidazoles in honey. Food Chem..

[20] Meng J., Wang Y., Zhou Y., Chen J., Wei X., Ni R., Liu Z., Xu F. (2020). A composite consisting of a deep eutectic solvent and dispersed magnetic metal-organic framework (type UiO-66-NH_2_) for solid-phase extraction of RNA. Microchim. Acta.

[21] Feng S., Zhang A., Wu F., Luo X., Zhang J. (2022). In-situ growth of boronic acid- decorated metal-organic framework on Fe_3_O_4_ nanospheres for specific enrichment of cis-diol containing nucleosides. Anal. Chim. Acta.

[22] Cassiano N.M., Lima V.V., Oliveira R.V., De Pietro A.C., Cass Q.B. (2006). Development of Restricted-access Media Supports and Their Application to the Direct Analysis of Biological Fluid Samples Via High-performance Liquid Chromatography. Anal. Bioanal. Chem..

[23] Bagheri N., Lawati H.A., Hassanzadeh J. (2021). Simultaneous Determination of Total Phenolic Acids and Total Flavonoids in Tea and Honey Samples Using an Integrated Lab on a Chip Device. Food Chem..

[24] Thornton A.W., Jelfs K.E., Konstas K., Doherty C.M., Hill A.J., Cheetham A.K., Bennett T.D. (2016). Porosity in Metal–organic Framework Glasses. Chem. Commun..

[25] Haque E., Lee J.E., Jang I.T., Hwang Y.K., Chang J.S., Jegal J., Jhung S.H. (2010). Adsorptive Removal of Methyl Orange From Aqueous Solution with Metal-organic Frameworks, porous Chromium-benzenedicarboxylates. J. Hazard. Mater..

[26] Si Y., Wang W., El-Sayed E.M., Yuan D. (2020). Use of Breakthrough Experiment to Evaluate the Performance of Hydrogen Isotope Separation for Metal-organic Frameworks M-MOF-74 (M=Co, Ni, Mg, Zn). Sci. China Chem..

[27] Gao Q., Lin C., Luo D., Suo L., Chen J., Feng Y. (2011). Magnetic Solid-phase Extraction Using Magnetic Hypercrosslinked Polymer for Rapid Determination of Illegal Drugs in Urine. J. Sep. Sci..

[28] Dzah C.S. (2014). Influence of Fruit Maturity on Antioxidant Potential and Chilling Injury Resistance of Peach Fruit (*Prunus Persica*) During Cold Storage. Afr. J. Food Agric. Nutr. Dev..

[29] Li T., Lu M., Gao Y., Huang X., Liu G., Xu D. (2020). Double Layer MOFs M-ZIF-8@ZIF−67: The Adsorption Capacity and Removal Mechanism of Fipronil and Its Metabolites From Environmental Water and Cucumber Samples. J. Adv. Res..

[30] (2005). Harmonised Tripartite Guideline: Validation of Analytical Procedures: Text and Methodology, Q2 (R1).

[31] Rambla-Alegre M., Esteve-Romero J., Carda-Broch S. (2012). Is it really necessary to validate an analytical method or not? That is the question. J. Chromatogr. A.

[32] Silva C.L., Pereira J., Wouter V.G., Giró C., Câmara J.S. (2011). A fast method using a new hydrophilic–lipophilic balanced sorbent in combination with ultra-high performance liquid chromatography for quantification of significant bioactive metabolites in wines. Talanta.

[33] Gómez-Mejía E., Rosales-Conrado N., León-González M.E., Madrid Y. (2019). Determination of phenolic compounds in residual brewing yeast using matrix solid-phase dispersion extraction assisted by titanium dioxide nanoparticles. J. Chromatogr. A.

[34] Fan S., Yang G., Zhang J., Li J., Bai B. (2020). Optimization of Ultrasound-Assisted Extraction Using Response Surface Methodology for Simultaneous Quantitation of Six Flavonoids in Flos Sophorae Immaturus and Antioxidant Activity. Molecules.

[35] Ćirić A., Prosen H., Jelikić-Stankov M., Durdević P. (2012). Evaluation of matrix effect in determination of some bioflavonoidsin food samples by LC–MS/MS method. Talanta.

[36] Deng H., Li X., Peng Q., Wang X., Chen J., Li Y. (2005). Monodisperse magnetic singlecrystal ferrite microspheres. Angew. Chem. Int. Ed..

[37] Shi Y., Qiu B., Wu X., Wang Y., Zhu J., Liu X., Zhao D. (2020). Drug Delivery System and in Vitro Release of Luteolin Based on Magnetic Nanocomposite (Fe_3_O_4_@ZIF−67). Micro Nano Lett..

[38] Jarrah A., Farhadi S. (2020). Encapsulation of K6p2w18O62 into Magnetic Nanoporous Fe_3_O_4_/MIL-101 (Fe) for Highly Enhanced Removal of Organic Dyes. J. Solid. State Chem..

[39] Ye S., Jiang X., Ruan L.W., Liu B., Wang Y.M., Zhu J.F., Qiu L.G. (2013). Post-combustion Co_2_ Capture with the Hkust-1 and MIL-101(Cr) Metal–organic Frameworks: Adsorption, Separation and Regeneration Investigations. Micropor. Mesopor. Mat..

[40] Rostamnia S., Xin H. (2014). Basic Isoreticular Metal–organic Framework (IRMOF-3) Porous Nanomaterial as a Suitable and Green Catalyst for Selective Unsymmetrical Hantzsch Coupling Reaction. App. Organomet. Chem..

