# Supplementary material for: Magnetic/Zeolitic Imidazolate Framework-67 Nanocomposite for Magnetic Solid-Phase Extraction of Five Flavonoid Components from Chinese Herb Dicranopteris pedata
Source: Molecules. 2023 Jan 10;28(2):702. doi: 10.3390/molecules28020702 (PMC9866763; doi:10.3390/molecules28020702)
Supplement: Supplementary file 1 [file molecules-28-00702-s001.zip › molecules-2080167-supplementary-final.pdf]

Supplementary Materials

Magnetic/Zeolitic imidazolate framework-67 nanocomposite for magnetic solid-phase extraction of five flavonoid components from Chinese herb *Dicranopteris pedata*

Zhiyang Feng [1], Jiaqing Zhu [2], Shen Zhuo [2], Jun Chen [2], Wenyi Huang [2], Hao Cheng [2], Lijun Li [2], Tai Tang [2] *, Jun Feng [2, 3] *

[1.] KingMed College of Laboratory Medicine, Guangzhou Medical University, Guangzhou 510182, China

[2.] Department of Medicine, College of Biological and Chemical Engineering, Guangxi University of Science and Technology, Liuzhou 545006, China

[3.] State Key Laboratory for Chemistry and Molecular Engineering of Medicinal Resources, Guangxi Normal University, Guilin 541004, China

* Correspondence: tangchao199800@163.com (T.T.); hxpfengjun@gxust.edu.cn (J.F.)

S1. Preparation of magnetic MOFs

Preparation of Fe_3O_4 @ MIL-101 (Fe)

Fe_3O_4@MIL-101 (Fe) were prepared according to a previous report with slight modifications [1]. 0.02 g of Fe_3O_4 NPs and 3.375 g of $FeCl_3·6H_2O$ were dissolved in DMF (20 mL) and sonicated for 10 min to obtain solution A. 1.031 g of terephthalic acid was also dissolved in DMF (20 mL) and sonicated for 10 min to obtain solution B. Solutions A and B were mixed and sonicated for 5 min, and then transferred to a 50mL autoclave with a PTFE liner, heated at 110°C for 20 h, and cooled to 25 °C. After separation under an applied magnetic field, the resulting product was washed three times with hot ethanol and subsequently dried in a drying oven at 60 °C for 2 h.

Preparation of Fe_3O_4@MIL-101(Cr)

Fe_3O_4@MIL-101 (Cr) were prepared according to a previous report with slight modifications [2]. 0.4 g of $Cr(NO_3)_3·9H_2O$ and 0.02 g of Fe_3O_4 NPs were dissolved in DMF (40 mL) to obtain solution A. 0.83 g of terephthalic acid was also dissolved in DMF (40 mL) to obtain solution B. Solution A and solution B were mixed and transferred to a 100 mL autoclave with a PTFE liner, heated at 220 °C for 16 h, and cooled to 25 °C. After separation under an applied magnetic field, the resulting product was washed three times with methanol and dried in a drying oven at 105 °C for 2 h.

Preparation of Fe_3O_4@ZIF-4

Fe_3O_4@ZIF-4 were prepared according to a previous report with slight modifications [3]. 0.02 g of Fe_3O_4 NPs and 5.446 g of imidazole were dissolved in DI water (40 mL) to obtain solution A. 0.0594 g of $Zn(NO_3)_2·6H_2O$) was dissolved in DI water (20 mL) to obtain solution B. The two solutions were mixed and stirred at 50 °C for 3 h to allow complete growth of the crystals on the surface of Fe_3O_4NPs, followed by 12 h aging. After separation under an applied magnetic field, the resulting product was washed three times with DI water and dried in a drying oven at 80 °C for 12 h.

Preparation of Fe_3O_4@IRMOF-3

Fe_3O_4@IRMOF-3 were prepared according to a previous report with slight modifications [4]. 3.75 g of $Zn(NO_3)_2·6H_2O$ and 0.75 g of 2-aminoterephthalic acid were dissolved in DMF (25 mL), followed by the addition of 0.02 g of Fe_3O_4 NPs. After mixing, the mixture was transferred to an autoclave with a PTFE liner and heated in an electric oven at 100 °C for 17 h. After separation under an applied magnetic field, the product was washed 3 times with $CHCl_3$ and dried in a drying oven at 60 °C for 2 h.

Preparation of Fe_3O_4@Zn-MOF-74

Fe_3O_4@Zn-MOF-74 were prepared according to a previous report with slight modifications [5]. 10.00 g of $Zn(NO_3)_2·4H_2O$ and 0.02 g of Fe_3O_4 NPs were dissolved in DMF (40 mL) to obtain solution A. 2.50 g of 2,5-dihydroxyterephthalic acid was dissolved in DMF (40 mL) to obtain solution B. After each solution was dissolved by sonication for 10 min, solution A and solution B were mixed and transferred to a 100mL autoclave with

a PTFE liner, heated at 100°C for 17 h, and cooled to 25 °C. After separation using an applied magnetic field, the resulting product was washed three times with methanol, dried in a drying oven at 60 °C, and then ground for subsequent experiments.

Preparation of Fe_3O_4@Co-MOF-74

Fe_3O_4@Co-MOF-74 were prepared according to a previous report with slight modifications [5]. 0.482 g of 2,5-dihydroxyterephthalic acid and 0.02 g of Fe_3O_4 NPs were dissolved in DMF (40 mL) to obtain solution A. 2.377g of Co $(NH_3)_2 \cdot 6H_2O$ was dissolved in DMF (40 mL) to obtain solution B. Solution A and solution B were mixed and transferred to a 100 mL autoclave with a PTFE liner and heated at 110 °C for 12 h. After separation using an applied magnetic field, the resulting product was washed three times with methanol, dried in a drying oven at 60 °C, and then ground for subsequent experiments.

Table S1. The gradient elution conditions of HPLC.

Time/min	A (%)	B (%)
0	20	80
24	50	50
25	20	80

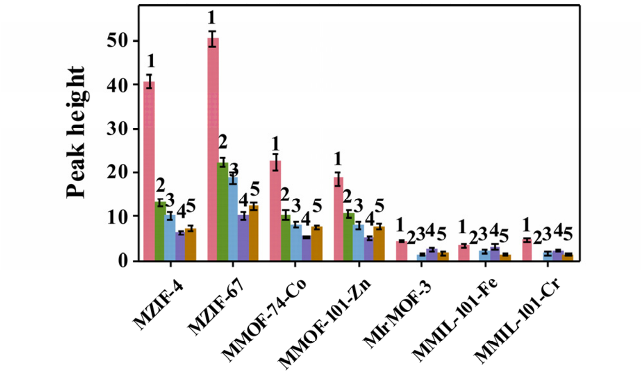

Figure S1. The extraction performance of Seven different magnetic MOFs.
1. Rutin; 2. Quercitrin; 3. Kaempferol-3-O-α-L-rhamnoside; 4. Quercetin; 5. Kaempferol.

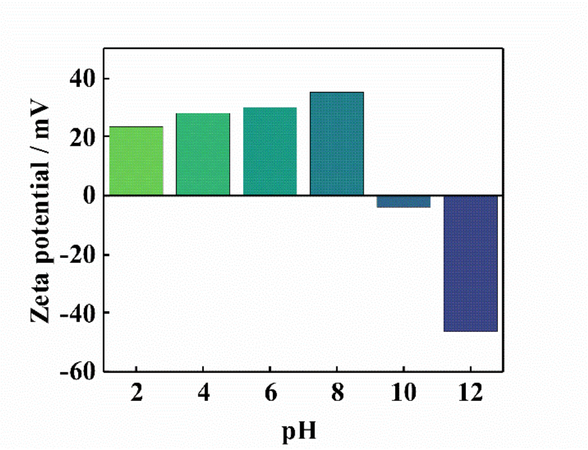

Figure S2. The zeta potential for different pH.

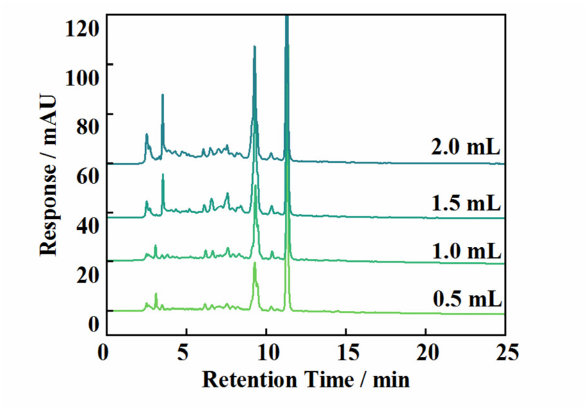

Figure S3. Chromatograms of *Dicranopteris pedata* samples after desorption with different desorbing solvent volume.

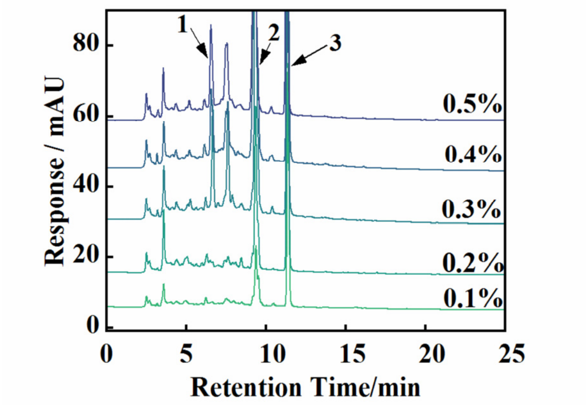

Figure S4. Chromatograms of *Dicranopteris pedata* samples after desorption with different desorbing solvent pH. 1. Rutin; 2. Quercitrin; 3. Kaempferol-3-O-α-L-rhamnoside.

References

1. Jarrah, A.; Farhadi, S. Encapsulation of K6p2w18O62 into Magnetic Nanoporous Fe_3O_4/MIL-101 (Fe) for Highly Enhanced Removal of Organic Dyes. *J Solid State Chem* **2020**, *285*, 121264.
2. Ye, S.; Jiang, X.; Ruan, L.W.; Liu, B.; Wang, Y. M.; Zhu, J. F.; Qiu, L.G. Post-combustion Co_2 Capture with the Hkust-1 and MIL-101(Cr) Metal–organic Frameworks: Adsorption, Separation and Regeneration Investigations. *Micropor Mesopor Mat* **2013**, *179*, 191-197.
3. Bagheri, N.; Lawati, H. A.; Hassanzadeh, J. Simultaneous Determination of Total Phenolic Acids and Total Flavonoids in Tea and Honey Samples Using an Integrated Lab on a Chip Device. *Food Chem* **2021**, *342,* 128338
4. Rostamnia, S.; Xin, H; Basic Isoreticular Metal–organic Framework (IRMOF-3) Porous Nanomaterial as a Suitable and Green Catalyst for Selective Unsymmetrical Hantzsch Coupling Reaction. *App Organomet Chem* **2014,** *28*, 359-363.
5. Si, Y.; Wang, W.; El-Sayed, E.M.; Yuan, D. Use of Breakthrough Experiment to Evaluate the Performance of Hydrogen Isotope Separation for Metal-organic Frameworks M-MOF-74 (M=Co, Ni, Mg, Zn). *Sci China Chem* **2020**, *63,* 881-889.
